# Patient‐specific cutting guides allow 1° precision in asymmetric anterior closing‐wedge osteotomy

**DOI:** 10.1002/jeo2.70131

**Published:** 2024-12-30

**Authors:** Julien Leluc, Ahmed Mabrouk, Jacob Hirth, Danyal Nawabi, Christophe Jacquet, Matthieu Ollivier

**Affiliations:** ^1^ Department of Trauma and Orthopaedics, Institute for Locomotion, Sainte‐Marguerite Hospital Aix‐Marseille University Marseille France; ^2^ Department of Trauma and Orthopaedics Basingstoke and North Hampshire Hospital Basingstoke UK; ^3^ Department of Trauma and Orthopaedics Hospital for Special Surgery New York New York USA

**Keywords:** ACL rupture, anterior closure osteotomy, cutting guide, precision, tibial slope, varus

## Abstract

**Purpose:**

Asymmetric anterior closing‐wedge high tibial osteotomy (ACWHTO) allows correction of both excessive posterior tibial slope (PTS) and varus deformity. However, the complexity of this surgery requires a high degree of accuracy, which is less likely to be achieved with standard instrumentations. This study aimed to determine the accuracy of 3D patient‐specific cutting guides (PSCGs) to provide the accurate planned correction in the frontal and sagittal planes.

**Methods:**

Eight sawbones tibiae were identically printed from the same patient data. An ACWHTO with a PSCG was performed on each sawbone. Postoperative measurements of PTS, mechanical medial proximal tibial angle (mMPTA), hinge area and hinge–posterior cruciate ligament (hinge–PCL) distance were compared with the preoperative planned measurements. The precision was defined as the absolute difference (∆) between the target planned values and postoperative values.

**Results:**

The mean accuracy was 0.6° ± 0.74° for PTS, 0.8° ± 0.71° for mMPTA, 0.3 ± 0.2 cm^2^ for hinge area and 0.1 ± 0.06 mm for hinge–PCL distance.

**Conclusion:**

In the setting of sawbones, the use of PSCGs was a reliable and accurate method of achieving simultaneous correction in the frontal and sagittal planes during asymmetric ACWHTO.

**Level of Evidence:**

Level V, basic science biomechanical laboratory study.

**Abbreviations**
ACL: anterior cruciate ligament
ACLR: anterior cruciate ligament reconstruction
ACWHTO: anterior closing‐wedge high tibial osteotomy
ICC: intraclass correlation coefficient
MPTA: medial proximal tibial angle
PCL: posterior cruciate ligament
PPTA: proximal posterior tibial angle
PSCGs: patient‐specific cutting guides
PSI: patient‐specific instrumentation
PTS: posterior tibial slope

## INTRODUCTION

Despite technical advancement and satisfactory results of anterior cruciate ligament reconstruction (ACLR), the re‐rupture rate following reconstruction remains high (10%–20%) [2, 33]. Failure of ACLR has been repeatedly attributed to a multitude of extrinsic factors [1, 8, 21, 26, 30]. Nevertheless, several studies have emphasized screening for the intrinsic risk factors and their role in ACLR failure [7, 11, 23, 24] such as young age [32], narrow intercondylar notch or spherical lateral femoral condyle [31], hyperlaxity [19] and knee malalignment in the coronal and/or the sagittal planes. In the coronal plane, a 5° of varus malalignment was demonstrated to be associated with increased ACL stress [15]. Similarly, in the sagittal plane, excessive posterior tibial slope (PTS) has been shown to increase anterior tibial translation and shear forces on the ACL [3, 6, 9, 20, 27, 29].

Correction of the tibial morphology, in both the coronal and sagittal planes, remains complex [12, 16, 22]. Medial opening‐wedge osteotomy is the gold standard to correct varus malalignment but has limited potential to reduce excessive PTS [16]. However, an anterior closing‐wedge high tibial osteotomy (ACWHTO) is an efficient method to correct steep PTS [14]. A potential solution to correct the proximal tibial geometry in both the coronal and sagittal planes is to perform an asymmetric ACWHTO. However, an asymmetric ACWHTO is a very demanding surgery. It is less likely to conclude to the desired level of correction in both planes, using the standard instrumentations.

Patient‐specific instruments (PSIs) may help surgeons achieve a precise biplanar correction of the proximal tibial morphology. The 3D patient‐specific cutting guides (PSCGs) have demonstrated high reliability and accuracy in correcting coronal plane malalignment through performing open‐wedge high tibial osteotomy [27]. This study aimed to determine the precision of these PSIs in achieving precise correction of both coronal and sagittal planes through performing asymmetric ACWHTO. We hypothesized that PSI could provide a 1° precision of correction in both the sagittal and coronal planes.

## METHODS

### Preoperative planning

**Figure 1:**
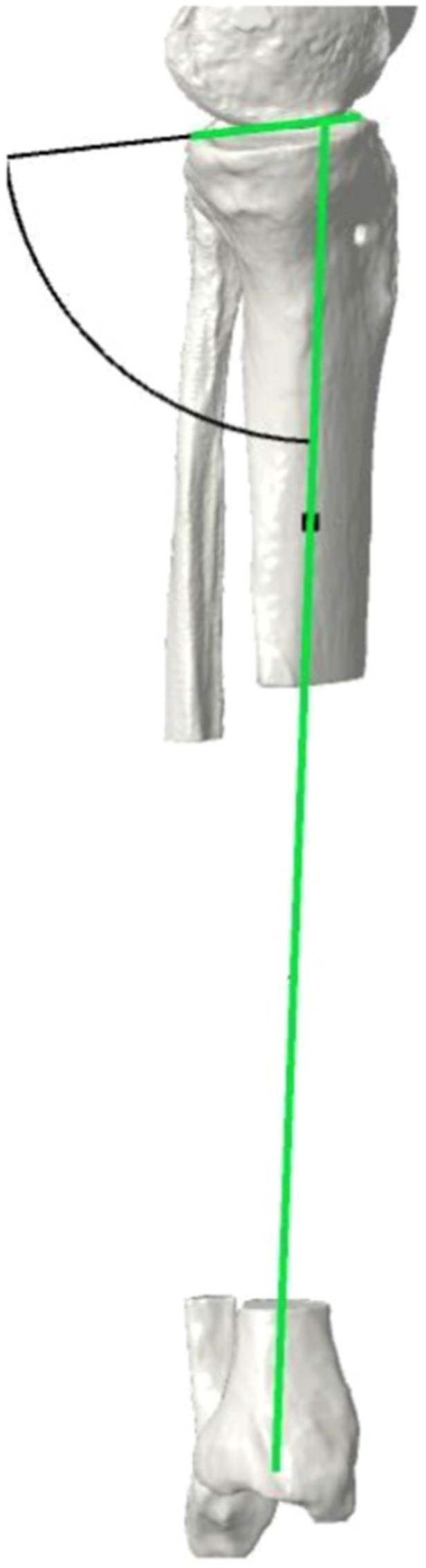
Angle measurements on 3D models. (a) Mechanical medial proximal tibial angle (mMPTA) measures the medial angle between the mechanical axis of the tibia and the tangent to the tibial plateau in the coronal plane. mMPTA = 81.6°. (b) Posterior tibial slope (PTS) is the posterior angle between the line orthogonal to the mechanical area and the tangent to the medial tibial plateau in the sagittal plane. PTS = 8.6°.

In this study, the left knee of a 45‐year‐old male patient was scanned using a standardized computed tomography (CT) protocol. The following acquisition parameters included 120 kV, 400 mA and 0.625‐mm‐thick slices. The DICOM images were then imported into Mimics software, and a 3D geometric model of the tibia was created. The bone was composed of plastic cortical shell and inner cancellous material. Anatomic landmarks, anatomic reference planes and the mechanical axis of the tibia were defined on the 3D model. The 3D planning tool was Materialize 3‐matic. The preoperative mechanical medial proximal tibial angle (mMPTA), which indicates the tibial deformity in the coronal plane, and the proximal posterior tibial angle, which indicates the PTS (sagittal plane deformity), were measured at 81.6° and 8.6°, respectively (Figure 1).

**Figure 2:**
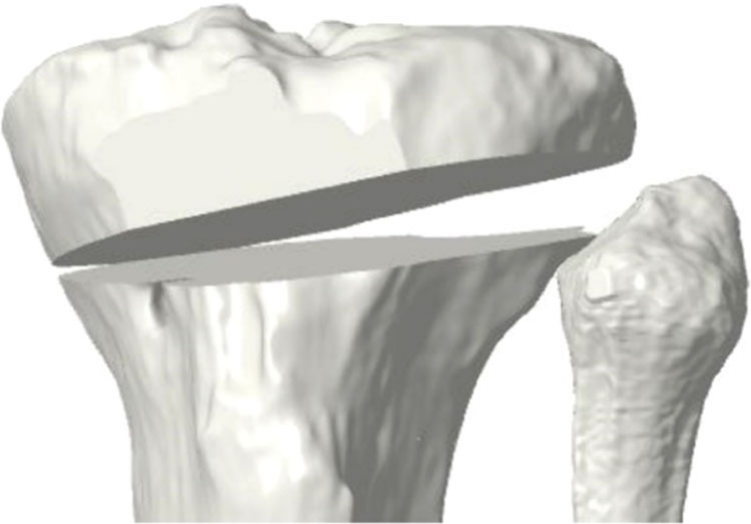
3D modeling of the asymmetric anterior closing‐wedge high tibial osteotomyduring preoperative planning.

**Figure 3:**
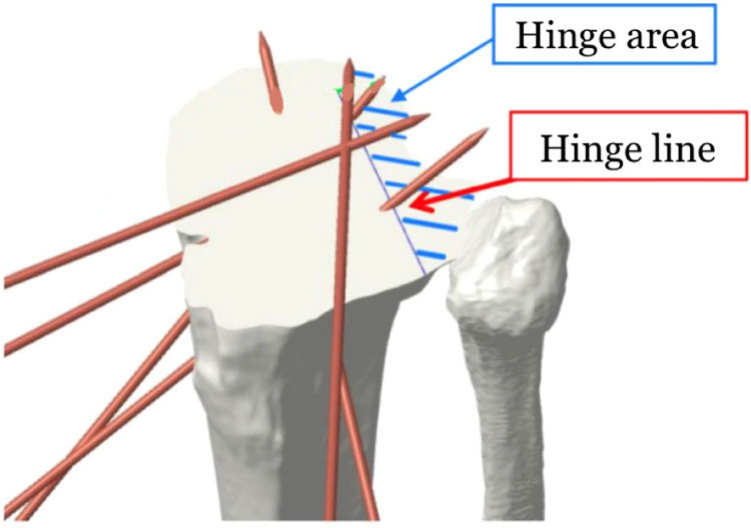
Representation of the hinge surface on a side superior view of the 3D model.

Eight sawbones tibiae were created from the patient‐specific DICOM and 3D model. Therefore, all the sawbones samples had identical morphology to the patient's tibia. An 8° correction was determined for the coronal plane deformity (mMPTA) and a 6° correction was determined for the sagittal plane deformity (PTS). The preoperative 3D tibia model was imported into a 3D planning tool. A cutting plane was positioned and the ACWHTO was simulated for the previously determined coronal and sagittal plane corrections (Figure 2). Additionally, the planned hinge area of the osteotomy and the distance between this hinge and the posterior cruciate ligament (PCL) insertion (hinge landmark [25]) were measured on the preoperative 3D model (Figure 3).

The cutting guides were composed of medial and lateral parts. The PSCGs were designed based on the simulation of the ACWHTO. The cutting guides considered the anatomy of the tibia, the position of the cutting plane and the amount of correction expected in all planes. All PSCGs were 3D printed.

### ACWHTO

**Figure 4:**
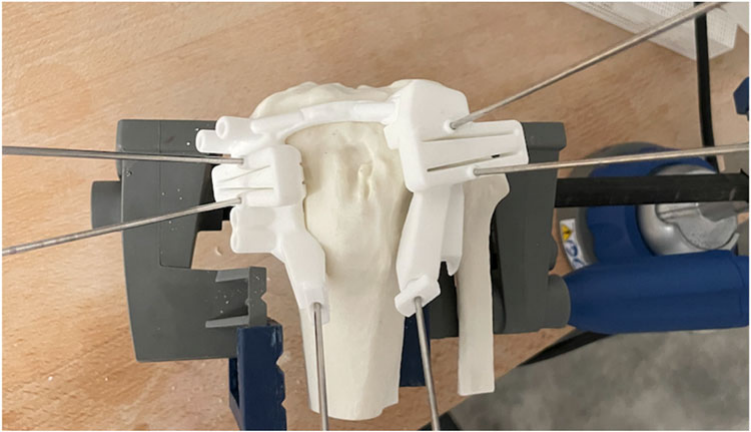
Anterior view of K‐wire placement on the sawbone model.

**Figure 5:**
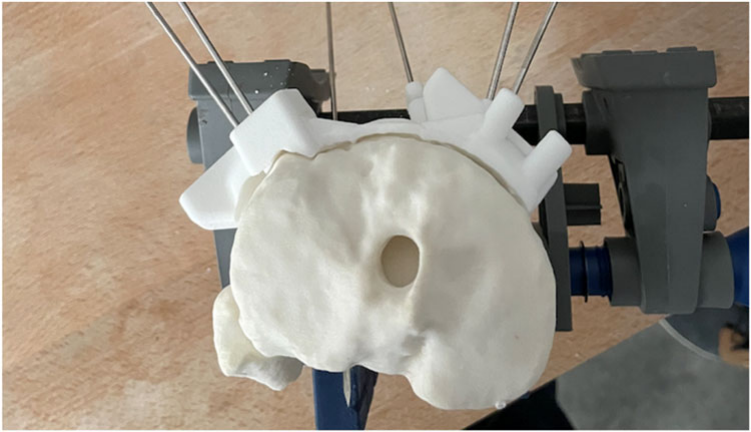
Top view of K‐wire placement on the sawbone model.

All ACWHTOs were performed by a single senior surgeon (M. O.), trained in performing osteotomies using PSCGs. The cutting guides were composed of medial and lateral parts. Three K‐wires were used to attach the cutting guides to the sawbone tibiae: two on the lateral part and one on the medial. Three K‐wires (two on the lateral part and one on the medial) were inserted to protect the osteotomy hinge and (virtual) posterior structures (Figures 4 and 5).

The same CT protocol was then used to analyze the tibiae after ACWHTO. Post‐ACWHTO 3D geometric models were created with Mimics software, where PTS, mMPTA, hinge area and PCL–hinge distance were measured. The precision was defined as the absolute difference (∆) between the target planned values and postoperative values.

### Statistical analysis

Statistical tests were performed using SPSS software by a trained statistician. A comparative statistical analysis between the preoperatively planned angles and the postoperative measurements of those angles was performed using nonparametric tests. The level of statistical significance was set at *p* < 0.05. Planned and postoperative measurements were performed by two observers on two separate occasions. Intra‐ and interrater reliability was calculated using intraclass correlation coefficient (ICC).

## RESULTS

The cutting guides provided an average precision of ∆ = 0.6 ± 0.7° in the sagittal plane and ∆ = 0.8 ± 0.7° in the coronal plane. The average precision of the hinge area and the hinge–PCL distance were ∆ = 0.3 ± 0.2 cm^2^ and ∆ = 0.1 ± 0.1 mm, respectively (Table 1).

**Table 1:** Comparison of planned values and postoperative results; preoperative values are expressed as average and postoperative values are expressed as average ± standard deviation.

Parameter	Planned preoperatively	Average postoperatively	*p* Values
PPTA (°)	3	3.4 ± 0.92	0.3
MPTA (°)	89	88.5 ± 0.93	0.1
Hinge area (cm^2^)	4.1	3.8 ± 0.2	0.0008
Hinge–PCL distance (mm)	0.4	0.4 ± 0.11	0.3

Abbreviations: MPTA, medial proximal tibial angle; PCL, posterior cruciate ligament; PPTA, proximal posterior tibial angle.

For mMPTA, PTS and hinge–PCL distance, the difference between postoperative measurement and preoperative planning was not statistically significant (*p* > 0.05). Intra‐ and interobserver reliability was high with ICC values ranging from 0.7 to 0.9.

## DISCUSSION

The principal finding in the presented study is the confirmation of the study hypothesis that using PSCGs in asymmetric ACWHTO can achieve the planned correction with a 1° precision in both the coronal and sagittal planes. The correction accuracy achieved in the presented study was 0.8° in the coronal plane and 0.6° in the sagittal plane.

Precision is a key factor for the success of osteotomy in ACL‐deficient knees. Both undercorrection and overcorrection will negatively impact the clinical outcomes. In medial opening‐wedge high tibial osteotomy, an undercorrection negates the purpose of performing the osteotomy, and persistent varus may result in no clinical improvements. On the contrary, an overcorrection in the coronal plane may result in excessive valgization of the mechanical axis of the tibia. Consequently, abnormal load distribution on the knee lateral compartment will ensue with resultant increased pain, functional limitations and progressive arthrosis [13]. However, in the sagittal plane, an excessive tibial slope reduction has been shown to cause a significant increase in posterior tibial translation [4] and consequently will increase the load and strain on the PCL. Furthermore, decreased PTS was demonstrated to be a risk factor for primary PCL injury [4]. Patients with a decreased PTS were shown to have increased PCL tears when compared with age‐ and sex‐matched controls with intact PCLs [4].

PSCGs have been demonstrated to help achieve highly accurate corrections for different types of osteotomies. Jacquet et al. [17], in opening‐wedge distal femur varization osteotomy, reported a significant improvement in the accuracy of corrections using PSCGs compared to a control group with standard instrumentations. In the coronal plane, the PSCGs group showed an accuracy of 0.4 ± 0.5 versus 3.95 ± 1.6 in the control group, and in the sagittal plane, 0.5 ± 0.6 versus 3.1 ± 1.8 in the control group (*p* < 0.001). A comparable accuracy was reported by Donnez et al. [10], in a prospective cohort study with a larger sample size (*n* = 71): 0.5° in the coronal plane and 0.4° in the sagittal plane.

Moreover, better accuracy was reported in a cadaveric study of ten open‐wedge high tibial osteotomy using PSCG: a precision of a 0.2° and a 0.1° in the coronal and sagittal planes, respectively, was achieved [10].

Surgical correction in both the sagittal and the coronal planes in a complex osteotomy such as asymmetric ACWHTO is more likely to result in less accuracy between the planned and the achieved corrections. However, PSCGs have demonstrated high reliability and improved accuracy to reach subdegree precision in these two planes. In our study, an average ∆ of 0.6 ± 0.74 ° in the sagittal plane and 0.8 ± 0.71 ° in the coronal plane was achieved. An additional advantage of this technique is the preplanned position of the plate, which obviates the chance of ACL graft fixation or interference with the tibial tunnel with one of the screws or otherwise compromising the osteotomy fixation by using shorter screws. Furthermore, the cutting guides incorporate a K‐wire slot that limits the posterior cut. Hence, it serves to protect the hinge, which is critical in protecting the popliteal structures and limits the risk of secondary displacement or nonunion [25].

Numerous studies highlighted the relationship between excessive PTS and higher stress on the ACL with consequent increased risk of rupture or re‐rupture [3, 6, 9, 20, 27, 29]. Nevertheless, the indications for PTS correction are not consensual, neither is the amount of PTS correction [5]. Several authors propose a tibial slope‐changing osteotomy after a second iterative ACL rupture associated with a PTS > 12° [25, 28]. Others propose this surgery for the first ACL revision with an excessively steep PTS, or when associated with other risk factors such as meniscal lesions [9], differential laxity > 10° on lateral viewsor a PTS > 15° [14].

The limitations of this study include the small sample size; only one patient was included from which the eight sawbone tibia models were created. This allows room for statistical bias in the interpretation of the results and their extrapolation. Another limitation is the lack of a control group; however, the indications for ACWHTO are limited and there are no studies with a large sample size for this kind of osteotomy. Furthermore, this study was not comparative; a control group consisting of tibiae operated on freehand would make it possible to evaluate the real gain in precision provided by the SPCG. No accuracy of correction provided by ACWHTO has been reported in the literature, either freehand or with a customized cutting guide. PSCGs have been shown to reduce the operating time for different osteotomies [17, 18], but elements were not assessed in this noncomparative study.

Future studies may focus on creating a model to study the PTS slope alteration on both the ACL and the PCL combined. This might help guide the indications for PTS correction as well as the required correction degrees. Further randomized clinical studies should be conducted to validate the experimental results and evaluate the risk–benefit ratio of preoperative CT scans, which have been shown to result in a reduction in surgical time.

## CONCLUSION

In the setting of sawbones, the use of PSCGs was a reliable and accurate method of achieving simultaneous correction in the frontal and sagittal planes during asymmetric ACWHTO.

## AUTHOR CONTRIBUTIONS

All the authors participated in the study's content and design and have reviewed and agreed with the manuscript's contents.

## CONFLICT OF INTEREST STATEMENT

The authors declare no conflicts of interest.

## ETHICS STATEMENT

The authors have nothing to report.

## Data Availability

All data are available upon request with formal authorization from the hospital.
